# Calcification response of a key phytoplankton family to millennial-scale environmental change

**DOI:** 10.1038/srep34263

**Published:** 2016-09-28

**Authors:** H. L. O. McClelland, N. Barbarin, L. Beaufort, M. Hermoso, P. Ferretti, M. Greaves, R. E. M. Rickaby

**Affiliations:** 1 Department of Earth Sciences, University of Oxford, South Parks Road, Oxford, OX1 3AN, UK; 2 CEREGE CNRS-IRD-Aix Marseille Univ, Avenue Louis Philibert, BP80 13545 Aix en Provence cedex 04, France; 3 Consiglio Nazionale delle Ricerche, Istituto per la Dinamica dei Processi Ambientali(CNR-IDPA), c/o Universita’ Ca’ Foscari Venezia, Via Torino 155, Mestre Venice I-30172, Italy; 4 Godwin Laboratory for Palaeoclimate Research, Department of Earth Sciences, University of Cambridge, Downing Street, Cambridge CB2 3EQ, UK

## Abstract

Coccolithophores are single-celled photosynthesizing marine algae, responsible for half of the calcification in the surface ocean, and exert a strong influence on the distribution of carbon among global reservoirs, and thus Earth’s climate. Calcification in the surface ocean decreases the buffering capacity of seawater for CO_2_, whilst photosynthetic carbon fixation has the opposite effect. Experiments in culture have suggested that coccolithophore calcification decreases under high CO_2_ concentrations ([CO_2_(aq)]) constituting a negative feedback. However, the extent to which these results are representative of natural populations, and of the response over more than a few hundred generations is unclear. Here we describe and apply a novel rationale for size-normalizing the mass of the calcite plates produced by the most abundant family of coccolithophores, the Noëlaerhabdaceae. On average, ancient populations subjected to coupled gradual increases in [CO_2_(aq)] and temperature over a few million generations in a natural environment become relatively more highly calcified, implying a positive climatic feedback. We hypothesize that this is the result of selection manifest in natural populations over millennial timescales, so has necessarily eluded laboratory experiments.

Coccolithophores are the modern ocean’s dominant calcifying phytoplankton and coccoliths, the distinctive calcite plates that they produce, have populated the fossil record for over 200 million years^1^,^2^. Biogenic calcification is an important climatic feedback^3^,^4^, and in most organisms is thought to be strongly affected by changes in the carbonate chemistry of seawater. In coccolithophores, calcite precipitation occurs inside the cell, so the site of calcite precipitation is buffered from the external environment and is subject to an unusually high degree of biological control. The most abundant modern species of coccolithophore are *Emiliania huxleyi* and *Gephyrocapsa oceanica*; bloom-forming members of the family Noëlaerhabdaceae (Haptophyta: Coccolithophyceae: Isochrysidales: Noëlaerhabdaceae). Laboratory culture experiments have shown that these two species tend to calcify less when carbon dioxide concentrations are higher^5^,^6^,^7^, although the trade off between DIC availability and pH appears to be critical^8^. Yet these observed responses each characterize a single genome, adapted to the modern environment and exposed to artificially manipulated conditions in the laboratory in the absence of genetic change (phenotypic plasticity). In nature, communities are genetically and phenotypically heterogeneous, and selective pressures lead to competition. Environments are dynamic, and changes in average conditions are subtle and prolonged. Sexual reproduction, which has never been observed in the laboratory in coccolithophores, genetically homogenizes a population within a biological species, whilst accelerating adaptation through propagation of the most beneficial combinations of alleles and removal of deleterious mutations via natural selection. To elucidate the role of these phytoplanktonic marine calcifiers in nature across geological history, their response must be studied on timescales of tens to tens of thousands of years, within natural populations, where evolutionarily relevant processes such as meiosis and syngamy may occur^9^,^10^, and where the rate and amplitude of environmental change is representative of the real world. Indeed a theoretical model has suggested that on such timescales the selective pressure of increasing [CO_2_(aq)] may have favoured the more heavily calcified forms of coccolithophore^11^.

An apparent dichotomy exists between the consensus view of phenotypic plasticity as observed in short-term experiments and the theoretical result of long-term evolutionary adaptation. However, experiments lasting hundreds of generations have shown that asexual coccolithophore populations have the potential to adapt in culture^12^,^13^ and large-scale surveys have revealed trends across spatial environmental gradients in nature^14^,^15^. The fossil record, by contrast, is an archive of information about ancient natural coccolithophore communities that responded to real environmental changes over geological timescales. The challenge is to extract meaningful information from this resource. Isolated coccoliths in deep-sea sediment are often the only remnants of ancient coccolithophores to survive geological time, so inferences about the physiology of coccolithophores that lived in the past must come from this evidence alone.

## Results

### Size-normalization of coccolith mass

**Figure 1:**
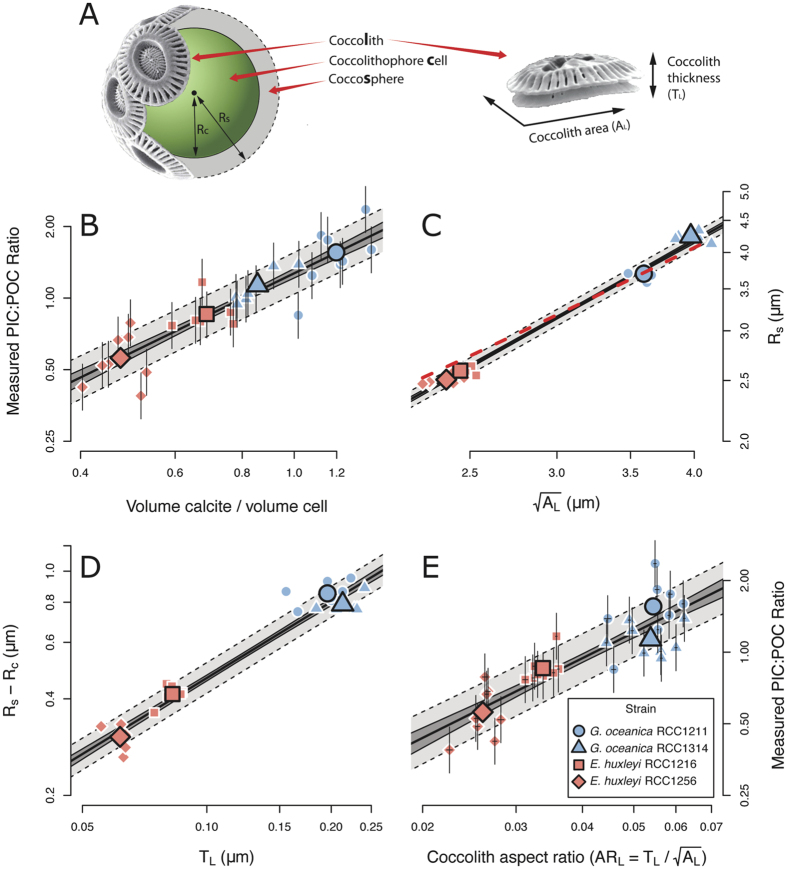
Size-normalisation of coccolith mass. (**A**) Schematic representation of coccolithophore cell, with variables defined. (**B)** Regression of molar PIC:POC ratio against volumetric ratios of calcite to organic material (Eq. 1). (**C**) Regression of coccosphere radius against the square root of coccolith area. The red dashed line represents an independently derived relationship between coccolith size and coccosphere size^21^. (**D**) Regression of coccosphere thickness against coccolith thickness. (**E**) Regression of molar PIC:POC ratio against coccolith aspect ratio (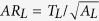; Eq. 3). The dark region around each regression line represents the 1*σ* confidence interval of the regression, whilst the lighter region with the dashed border represents the 1*σ* prediction interval of the regression. Error bars on individual points represent the 1*σ* confidence interval of each measurement. SEM images courtesy of Jeremy Young, used and adapted with permission (http://ina.tmsoc.org/Nannotax3/).

Studies to date have yielded contradictory results regarding the response of calcification to environmental change on geological timescales^14^,^16^, but this disagreement is, at least in part, a result of the lack of consistency between the parameter measured, and how this is inferred to represent “calcification intensity”. Coccolith mass has been used extensively to infer calcification ability^14^,^15^,^16^,^17^,^18^,^19^, but as it is not independent of coccolith size, size-normalization is highly nontrivial and it remains a biologically abstract quantity. In order to solve this problem, we have developed a procedure for size-normalising coccolith mass, by correlating an index based on coccolith morphometry with the molar ratio of particulate inorganic to particulate organic carbon (PIC:POC) of the biomass. The PIC:POC ratio is a direct record of the ratio of integrated rates of calcification to photosynthesis^3^, and therefore describes an energetic and carbon budget trade off between calcification and biological (metabolic) activity that makes more sense biologically than coccolith mass in the context of physiology and adaptation, and is independent of coccolithophore size. Through size-normalising mass by consideration of a dimensionless but biologically meaningful parameter, we circumvent problems associated with allometry. The concept for size-normalising coccolith mass is based on the following arguments:

- The molar PIC:POC ratio of coccolithophore biomass is proportional to the ratio of spherical volumes of calcite to organic matter (Fig. 1B).
- The square root of coccolith area (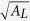), which we use as a 1-dimensional measure of coccolith size, is proportional to coccosphere radius (*R*_*s*_): 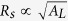 (Fig. 1C).
- Coccolith thickness (*T*_*L*_) is proportional to coccosphere thickness (*T*_*s*_ = *R*_*s*_ − *R*_*c*_): *T*_*s*_ ∝ *T*_*L*_ (Fig. 1D).
- Given (i), (ii) and (iii), a combination of coccolith mass and area can be related back to coccolithophore PIC:POC, via cell and coccosphere dimensions, thus providing a rationale for size-normalisation of mass (Fig. 1E).

We tested each assumption and calibrated our mass-normalisation concept using cultured coccolithophores. We grew two strains each of *E. huxleyi* and *G. oceanica* in artificial seawater, which was chemically altered to four different DIC concentrations, at constant pH, temperature and nutrient levels, in duplicate (see methods). (i) was established via *in vivo* measurement of coccosphere and cell volumes, and direct measurement of the molar PIC:POC ratio of the biomass:


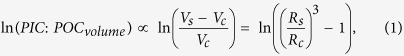


where *V*_*s*_ and *V*_*c*_ are respectively the solid spherical volumes of the coccosphere and the cell, and *R*_*s*_ and *R*_*c*_ are respectively the radii of the coccosphere and the cell (as labelled in Fig. 1A). Equation 1 is shown in Fig. 1B.

To generate disarticulated coccoliths, the filtered culture residue was bleached to remove organic matter whilst preserving the calcite intact, emulating the effect of decomposition. We analyzed this synthetic coccolith-sediment using SYRACO^20^; an automated image analysis tool, which measures coccolith area and mass (see methods). Our data support assumptions (ii) and (iii) (Fig. 1C,D respectively). The relationship that we find between coccolith size and cell size agrees with the well established relationship between coccolith length and cell size in the Noëlaerhabdaceae^21^, which is derived from fossils (red dashed line Fig. 1C). That our strains all lie on the same line as fossil Noëlaerhabdaceae supports the theory that this family of coccolithophores are a continuum of forms^14^,^21^. Equation 1 can be simplified by approximating the cell and coccosphere as having the same radius (*R*_*s*_). The volume of organic material is then the volume of a sphere of radius, *R*_*s*_, and the volume of calcite in the coccosphere is that of a thin shell of thickness *T*_*s*_ and radius *R*_*s*_. Using assumptions (ii) and (iii) gives:


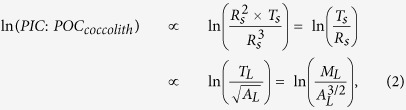


where *M*_*L*_ = coccolith mass, and *A*_*L*_ = coccolith area are the direct output from the SYRACO analysis, and *M*_*L*_/*A*_*L*_ = *T*_*L*_. As per (iv), an ordinary least squares linear regression incorporating an estimate of the uncertainty in PIC:POC based on the 1*σ* prediction interval gives:


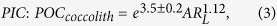


where 
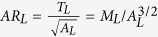
 is the lateral cross-sectional aspect ratio of a coccolith (AR_*L*_).

Equation 3 is derived from first principles, and is supported empirically. The calibration is shown in Fig. 1E. AR_*L*_ is taken to be the most sensible way of size-normalising coccolith mass, which alone is biologically meaningless. Caveats associated with directly translating AR_*L*_ to PIC:POC using Eq. 3 are discussed later.

### Down-core experiment

**Figure 2:**
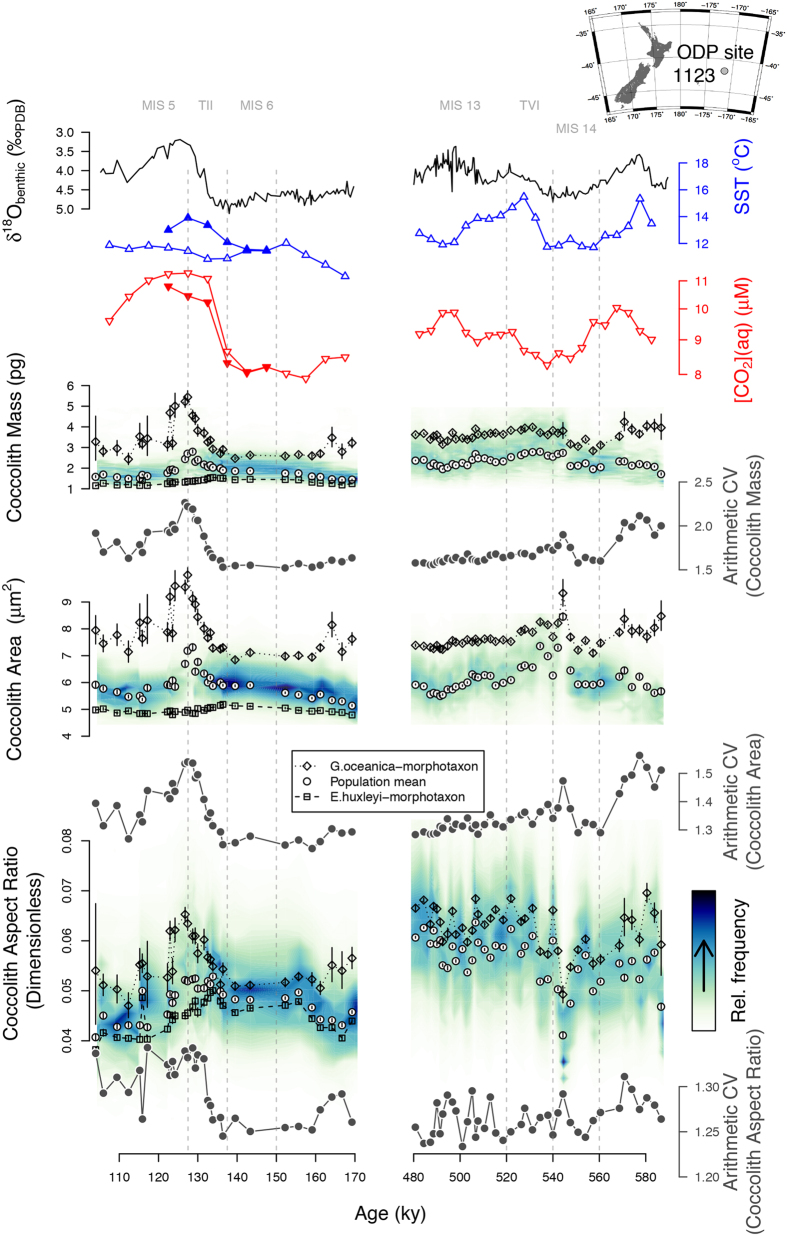
Calcification response of the Noëlaerhabdaceae to environmental changes over two glacial-interglacial cycles. (**A**) benthic *δ*^18^*O*^49^, and 5kyr interval average sea-surface temperature (from Mg/Ca ratios in planktic forams; filled triangles over TII represent an alternative size fraction - see methods) and [CO_2_(aq)] (calculated from CO_2_ atm^52^ and SST) at ODP site 1123 in the southern Pacific Ocean. (**B–D**) Coccolith morphometrics: (**B**) Mass, (**C**) Area and (**D**) Aspect ratio. Raw data are displayed as frequency-density contour plots. *Emiliania huxley*-affiliated morphotaxa (MG_*Emi*_) are hollow squares, *Gephyrocapsa* spp.-affiliated morphotaxa (MG_*Geo*_) are hollow diamonds and filled circles (population mean) represent the mean of the Noëlaerhabdaceae-affiliated morphotaxa in each sample. Arithmetic coefficient of variation (CV) is a measure of the mean-independent variance of log-normal data, and is shown in grey. Map was made using GMT 5.2.1 (http://gmt.soest.hawaii.edu/)^58^.

To explore the calcification response of natural coccolithophore populations to climatic change in the form of a down-core experiment, we used this novel approach to analyze sediment-core material from two glacial terminations, which are of contrasting magnitude (Fig. 2). The larger of these terminations sees an increase in atmospheric CO_2_ mixing ratios (CO_2*atm*_) similar in magnitude to that since the industrial revolution, albeit two orders of magnitude slower. SYRACO analysis was performed on sediment from ODP site 1123, on Chatham rise, east of New Zealand in the southernmost Pacific (41°47.2′S, 171°29.9′W, 3290 m water depth). Depths span the penultimate and 6th most recent glacial-interglacial cycles (MIS 7-5: ~200–100 ka and MIS 15-13: ~600–500 ka, respectively), and sample resolution is higher (~1000 year) over the terminations (TII: ~140–125 ka and TVI: ~540–520 ka). Dissolution proxies^22^ and SEM observations of coccoliths show good preservation at this site throughout the periods of interest. ODP site 1123 exists at the northern edge of the sub tropical front (STF) in the southernmost Pacific Ocean, and may see an increased influence of sub antarctic water during glacials and subtropical water during interglacials^22^. We used *in situ* proxy reconstructions of sea surface temperature (SST) and relative nutrient concentration to infer surface ocean conditions throughout the periods of interest. We calculated [CO_2_(aq)] from published CO_2*atm*_ records from ice cores (see methods) and from SST inferred from planktic foram Mg/Ca ratios (see methods), assuming equilibrium between the atmosphere and surface waters. [CO_2_(aq)] and temperature increased during each glacial termination, with no obvious change in relative nutrient concentrations (see methods and Fig. S2). pH and [DIC] were unconstrained, but on these timescales, the decrease in pH due to invasion of CO_2_ may be partially buffered by dissolution of carbonate sediments. For this reason, [CO_2_(aq)] is used to describe the carbonate system, as this is the most well constrained component.

SYRACO analysis^20^ measures the mass and area of each coccolith belonging to the family Noëlaerhabdaceae in the analysed samples. AR_*L*_ is calculated subsequently. Additionally, each coccolith is assigned to a morphotaxonomic group on the basis of shape. These groupings approximate species-level taxonomic classifications and provide insight into subsets of the population. Here we show morphotaxon-specific records for the groups dominated by large *Gephyrocapsa* spp. and by *E. huxleyi* (MG_*Geo*_ and MG_*Emi*_ respectively). These species are the most thoroughly studied in the laboratory, and their ultrastructure is suited to SYRACO analysis.

### Down-core results

Over TII, mean Noëlaerhabdaceae coccolith mass and area increase transiently across the termination. This is accompanied by an increase in the mean-independent variance (arithmetic coefficient of variation; ACV) in both of these variables (Fig. 2), implying a non-uniform response within the population. Specifically, the mass and area of MG_*Geo*_ transiently increase in parallel (almost doubling) over the termination, but (MG_*Emi*_) shows a subtle decrease. Over TVI, prior to the first appearance of *E. huxleyi* coccoliths in the fossil record at around 290 ka^23^, the absolute magnitude and ACV of Noëlaerhabdaceae coccolith mass is very stable, though with a large decrease in ACV during the MIS14 glacial inception. The absolute magnitude of coccolith area, however, peaks during MIS14 glacial maximum and decreases during TVI, but with little change in the area ACV. The time series of population mean AR_*L*_ (size-normalised mass) exhibits an increase across both terminations. This effect is paralleled but larger in the AR_*L*_ of MG_*Geo*_, which increases by ~30% across both TII and TVI. By contrast, the AR_*L*_ response of MG_*Emi*_ across TII is of a slight decrease. The absence of *E. huxleyi* explains the higher population mean AR_*L*_ over TVI than over TII, and its presence and opposing response to *G. oceanica*, as inferred from the morphotaxa, explains the increased variance observed over TII. The parameter AR_*L*_ reconciles the differing response of mass and area of MG_*Geo*_ to increasing [CO_2_(aq)] and temperature across these two terminations.

## Discussion

The AR_*L*_ time series implies that coccoliths become more calcified in Noëlaerhabdaceae, and especially *G.oceanica*, with increasing [CO_2_(aq)] and temperature at site 1123 in natural populations over a timescale of thousands of years. This increase contrasts with results from the literature that describe the capacity for phenotypic plasticity in these organisms. In the laboratory, single strains of *G. oceanica* calcify relatively less with increasing [CO_2_(aq)]^5^,^6^,^7^,^24^, and there is no discernible effect of temperature^7^. *E. huxleyi*’s plastic response to increasing CO_2_ concentration over the reconstructed [CO_2_(aq)] range of our time series is of decreasing calcification when [DIC] or alkalinity (ALK) is held constant^5^,^6^,^7^,^25^,^26^ but the opposite at constant pH^26^. In *E. huxleyi* the plastic effect of temperature in the laboratory is large, but non-linear and poorly constrained^7^.

**Figure 3:**
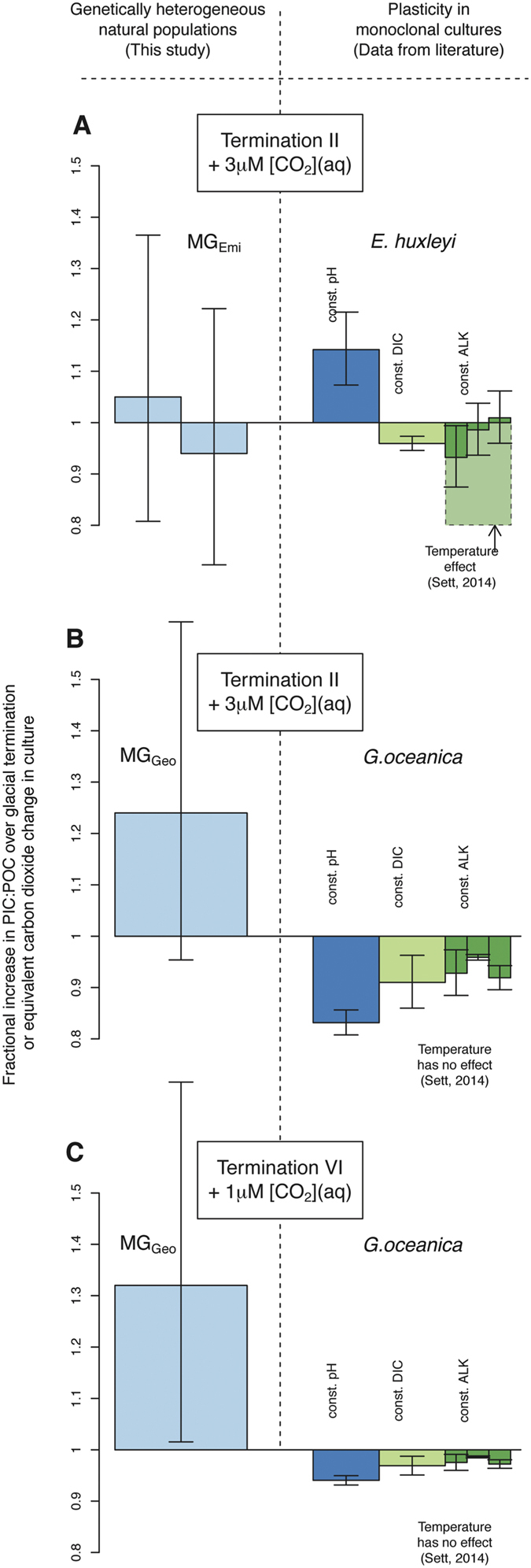
Fractional change in PIC:POC of natural heterogeneous populations over glacial terminations (left), compared with that of monoclonal strains subject to an equivalent CO_2_ change in culture (right). (**A**) The response of MG_*Emi*_ in nature is within the range of uncertainty and of plasticity of *E. huxleyi* observed in the laboratory. (**B,C**) The PIC:POC of MG_*Geo*_ in nature increases across both terminations, which is opposite to the equivalent plastic response as inferred from culture manipulations in *G. oceanica*. Error bars represent the 1*σ* uncertainty, which for the down-core response, is calculated using the prediction interval of Eq. 3. For culture results, details of the regressions are given in the Supplementary material.

So far, our proposed method for size-normalising coccolith mass has used the PIC:POC ratio simply to justify our theoretical rationale with real biological data. It is, however, possible to use this relationship as a proxy for PIC:POC, using the coccolith aspect ratio as an input. The predictive power of Eq. 3 as a proxy for Noëlaerhabdaceae PIC:POC is dictated by the spread of the residuals about the regression line: the prediction interval - not only by the confidence interval, which describes the uncertainty in the relationship^27^. With due consideration of the associated caveats, and formal consideration of uncertainty, Eq. 3 can be used as a proxy to directly infer PIC:POC from Noëlaerhabdaceae coccolith morphometry (Fig. 3). If the constant of proportionality between coccolith size and cell size is systematically influenced by the environment or differences between species, this will be reflected in the estimate of PIC:POC. In our calibration, hundreds of thousands of cells, and thousands of individual coccoliths were measured for each sample, which has the effect of averaging over the potential variation in the slope of Eq. 3 due to variations in the number of coccoliths per cell, or the degree of overlap of coccoliths. Indeed, the established relationship between coccolith size and cell size in heterogeneous natural fossil populations^21^ agrees with ours very well (Fig. 1C), lending support to the constant relationship between cell size and coccolith size, and application to the whole Noëlaerhabdaceae family. Coccolith over-production in *E. huxleyi*, the only species where multiple coccolith layers are produced, will cause coccolith morphometry to underestimate net PIC:POC, and this is unavoidable. In the rare deposits where preservation is exceptional and coccospheres are intact, such as in the Lagerstätte deposits of Tanzania^28^,^29^, Eq. 1 may be used to estimate PIC:POC directly. This approach would bypass the assumptions necessary to infer PIC:POC directly from disarticulated coccoliths.

With the aforementioned caveats in mind, changes in AR_*L*_ can be used to directly estimate the PIC:POC ratio of the biomass in these ancient organisms using Eq. 3, albeit with a rather large associated uncertainty. We have compiled PIC:POC values from the literature and compared them with results inferred from our down-core record (Fig. 3 and Fig. S1). The PIC:POC of both the Noëlaerhabdaceae family mean, and of MG_*Geo*_, increases in response to [CO_2_(aq)] and temperature. This is opposite to results from the laboratory (Fig. 3). Any change in PIC:POC inferred from AR_*L*_ of MG_*Emi*_ coccoliths are well within the range of uncertainty however. Although the uncertainties in these estimates are large, these results are necessarily elusive to laboratory experiments due to the timescales involved.

**Figure 4:**
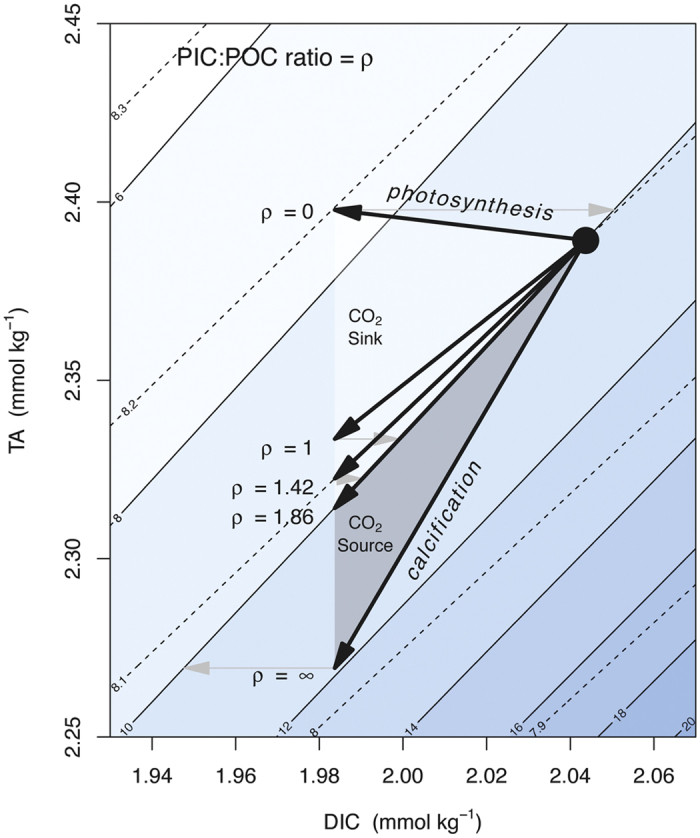
Instantaneous effect of biomass production on seawater carbonate chemistry as a function of PIC:POC (*ρ*). Solid line isocontours represent [CO_2_] (*μ*mol/kg), and dashed isocontours represent pH. Solid arrows represent the instantaneous effect of biogenic matter formation, and shaded arrows CO_2_ exchange with an atmospheric carbon pool that is large relative to the perturbed sample of seawater. Depending on its *ρ*, biogenic material may form an instantaneous sink or source of CO_2_. For conditions typical of the modern ocean, when *ρ* is <or > 1.42, pH initially increases or decreases respectively, and when *ρ* is < or > 1.86 [CO_2_] initially decreases or increases respectively. These critical values depend on the carbonate chemistry of the surface ocean^59^.

Phenotypic plasticity, as observed in laboratory experiments to date, cannot explain coccoliths becoming more calcified with increasing [CO_2_(aq)] and temperature. Based on our down-core observations we infer, therefore, either a component of selection for more heavily calcifying forms, or that the parameter space, and thus phenotypic plasticity, has not been thoroughly explored in the laboratory. Selection may drive evolution of the population internally through differential suitability of forms to the abiotic environment, or through differential susceptibility to grazers or viral attacks^30^, or via propagation of differentially adapted forms introduced from elsewhere along gradients of nutrients, pH, salinity or temperature. Alternatively the apparent increase in size-normalised coccolith mass, within MG_*Geo*_ in particular, may be due to a transient increase in the relative abundance of established more highly calcifying pseudo-cryptic sub lineages^14^,^31^. The increased mean-independent variance in size-normalised coccolith mass across TII may be the result of selection for different growth strategies, and/or differences in phenotypic plasticity across the population. As a substrate, low CO_2_ concentrations may limit growth rate^32^, so increasing its concentration may allow for faster growth; but elevated CO_2_ at constant alkalinity decreases the saturation state of calcite making it more energetically costly to calcify. Increases in CO_2*atm*_ also drive increases in temperature. To explain the increased variance over TII by the differential effect of phenotypic plasticity within the population superimposed on a general selection for more highly calcified forms, the more lightly calcifying forms would need to be more susceptible to a decreasing saturation state, but this is not observed in culture. If anything, the relatively heavily calcifying *G. oceanica* appears to exhibit a greater plastic decrease in size-normalised coccolith mass with increasing [CO_2_(aq)] than the relatively lightly calcifying *E. huxleyi*. Alternatively, increasing [CO_2_(aq)] could conceivably create diverging niches; lightly calcifying forms could be selected on the basis of faster growth on a given CO_2_ substrate, and highly calcifying forms could be selected on the basis of reduced mortality due to the greater integrity of their calcitic (presumably defensive) structure. Whatever the mechanism, it is interesting to note that the size-normalised coccolith mass of the population is driven to a very narrow range during glacial times (Fig. 2). If this observation does indeed reflect a narrow range of PIC:POC values, it may indicate that it is universally energetically favourable, at low temperature and [CO_2_(aq)], to maintain a balance between the relative rate of photosynthesis to calcification, thus minimizing the effect of biomass production on intracellular carbonate chemistry (Fig. 4).

From a biogeochemical viewpoint, calcification and photosynthesis alter the buffering capacity of water for CO_2_ in opposing ways through their respective effects on [DIC] and [ALK]. These effects are relevant on scales ranging from the sub-cellular environment in coccolithophores to global biogeochemical cycles. In the sub-cellular environment, the ratio of calcification to photosynthesis determines the steady state drift in intracellular pH, that would otherwise occur if it were not counteracted by the active pumping of protons into or out of the cell (Fig. 4). During blooms, coccolithophores constitute an instantaneous net sink or source of CO_2_, or drive a change in pH, depending on their PIC:POC ratio (Fig. 4). Under typical modern conditions, formation of biomass in the surface ocean with a PIC:POC of higher or lower than ~1.86 constitutes respectively an instantaneous source or sink of CO_2_ (Fig. 4), with this critical value varying with the carbonate chemistry of the water. This effect is not permanent however; the effect of biomass production on surface ocean seawater chemistry is often transient due to respiration and remineralization of organic matter by grazers, and dissolution of calcite causing cycling within the surface ocean. Carbon fluxes between the atmosphere and ocean are strongly influenced by export of organic matter and calcite from the surface ocean. Export has a prolonged effect on surface ocean/atmosphere partitioning of CO_2_ because DIC and ALK are sequestered in the deep ocean or sediments, which have residence times of respectively hundreds, and tens of thousands of years. Coccolithophores are responsible for up to half of the calcification in the surface ocean^1^, with members of the family Noëlaerhabdaceae typically contribute around half 33. It is through their role in pumping calcite to depth that coccolithophores constitute a significant lever on the global climate system.

In the modern ocean, phytoplankton stock is a function of light, nutrients, grazing and viral lysis^34^. The effect of predators and viruses cannot explain the current distribution of primary production in the surface ocean, which is dominated by nitrate and iron availability^35^. In coccolithophores, the PIC:POC ratio describes the amount of calcite produced per unit organic matter. As organic matter production is finite and limited by the availability of biologically accessible nitrogen and iron, the PIC:POC ratio directly corresponds to the amount of calcite produced for a given standing stock or flux of ultimately limiting nutrients. In the absence of other changes, an increase in the PIC:POC ratio of coccolithophores in the surface ocean leads to an increase in the ratio of ALK:DIC exported, and thus a decrease in the buffering capacity of the surface ocean for CO_2_. A confounding effect arises because cells are approximately the same density as water, and must be ballasted by heavy minerals, including calcite, in the form of aggregates or faecal pellets in order to be exported from the surface ocean before being remineralised. In terms of the net effect on seawater carbonate chemistry in the surface ocean, export of calcite may therefore constitute a trade-off between direct removal of calcite, and the effect this has on the rate of export of organic matter^36^.

In our down core record, coccolithophores belonging to the family Noëlaerhabdaceae appear, on average, to calcify more under increasing [CO_2_](aq) and temperature. Although it is impossible to decouple the effects of temperature, [CO_2_(aq)], salinity and nutrient availability down-core, these parameters have varied together throughout geological time. On glacial-interglacial timescales therefore, to first order, the Noëlaerhabdaceae’ may actually constitute a positive feedback to increasing CO_2*atm*_ on millennial timescales.

We have shown that natural coccolithophore populations appear to adapt to rising [CO_2_(aq)] and temperature on a millennial timescale, dominantly via selection for an increased tendency to calcify. Thus, this work introduces a temporal dimension to the prevailing view based on the results of culture manipulation experiments. The theoretical model predicting this outcome describes a trade-off between fast growth and calcification^11^, confounding the implications for the total rate of calcite production in the surface ocean. We anticipate our results to be of use to biogeochemical modellers, but a more thorough understanding of the fate of biogenic material produced in the surface ocean is essential before the full implications of this work are realised.

## Methods

### Culture experiments

Duplicate monoclonal batch cultures of four strains of coccolithophore belonging to the family Noëlaerhabdaceae were grown in sterile filtered (0.2 *μ*m) artificial seawater prepared according to ESAW^37^ adapted for a range of DIC concentrations ([DIC] = 1.380 mM, 2.147 mM, 3.067 mM and 6.135 mM) at constant pH (8.2) by varying sodium bicarbonate addition and titration with HCl and with nitrate (442 *μ*M), phosphate (5.00 *μ*M), vitamins, trace metals and EDTA according to *K*/2^38^. Carbonate chemistry manipulation at constant pH is more analogous to changes expected in the surface ocean on a glacial-interglacial timescale than holding alkalinity constant, due to buffering by carbonate sediments. Cultures were maintained at 15 °C with an incident photon flux of 250 *μ*E and a 12/12 light/dark cycle. Cells were acclimated for >20 generations in dilute batch culture for each experimental condition prior to inoculation. Cells were inoculated in 2.4 l polycarbonate flasks, with no headspace and sealed off to the air with teflon lined caps. Removal of medium during the experiment was unavoidable due to the need to count and measure cells, and resulted in a maximum headspace of 20 cm^3^ at harvest. In order to minimise the drift in culture conditions throughout the course of the experiment, cells were harvested at ~1–2% (and never greater than 4%) of maximum cell density, which was determined for each experimental condition and strain combination via preliminary experimentation. Strains were AC478 (RCC1211 *Gephyrocapsa oceanica* from Portuguese coast in Atlantic Ocean), AC472 (RCC1216 *Emiliania huxleyi*, from Tasman Sea in Pacific Ocean), AC448 (RCC1256 *Emiliania huxleyi*, Icelandic coast in Atlantic Ocean) and AC279 (RCC1314 *Gephyrocapsa oceanica*, French coast in Atlantic Ocean) from the Roscoff culture collection (RCC). Particulate material was harvested by dry filtration onto pre-weighed membranes with 0.2 *μ*m pore-size, and rinsed of salt with a minimal amount of deionised water (adjusted to pH 7). Coccolithophore size and concentration were obtained using a Beckman Z2 Coulter Counter (see ref. 39 for description of Coulter principle). Coccosphere and cell size were measured three times each respectively pre- and post-decalcification both morning and evening on the harvest day and the preceding day. Cells were decalcified by reducing the pH of the suspension with HCl addition to 5.0 with for around 20 minutes. The Coulter counter was calibrated to use ESAW + *K*/2 medium as an electrolyte, and for use with the acidified electrolyte, to accommodate for the difference in ionic strength. Cell division is synchronized under the light/dark cycle and cell size was assumed to increase linearly throughout the day^40^. By measuring cell and coccosphere size morning and evening, the bias introduced due to the time of day of measurement can be removed by interpolation to the same time of day. This also removes the daily variation in the slope of Eq. 3 which is a function of cell size^40^ and number of coccoliths per cell. Culture health was monitored by cell counts and microscope inspection on alternate days. Molar PIC and POC were measured with a *Rock Eval analyser*, which is preferable to making assumptions about carbon density of biogenic material. An aliquot of culture residue was bleached with dilute sodium hyperchlorite solution (4% available chlorine for 20 minutes) to remove the organic matter, and washed three times in deionised water to remove the bleach. The resultant “pseudo-sediment” was subsequently analysed using the computational software, SYRACO^20^,^41^,^42^.

### Stable isotope and Mg/Ca measurements on planktonic foraminifera

Paired stable isotope (*δ*^18^*O* and *δ*^13^*C*) and Mg/Ca analyses were performed on typically 60 individual shells of *Globigerina inflata* (MIS 15-13) and *Globigerina bulloides* (MIS 7-5), picked from the 300–355 *μ*m size fraction. In Fig. 2, the alternative filled points are temperatures inferred from the 250–300 *μ* size fraction, which captures the glacial termination more clearly than the larger fraction, but has little effect on [CO_2_(aq)]. Prior to isotopic analyses, samples were crushed, cleaned in 3% hydrogen peroxide solution to remove any possible organic contaminants, rinsed with acetone and dried overnight in an oven at 60 °C. Measurements of the isotopic composition of carbon dioxide, released from the foraminiferal carbonate using a MULTIPREP system, were performed on a VG SIRA mass spectrometer at the Univ. Cambridge. Calibration to the Vienna Peedee Belemnite standard was through the NBS19 standard^43^, and the analytical precision was better than 0.08 for *δ*^18^*O* and 0.06 for *δ*^13^*C*. For Mg/Ca measurements, samples were prepared following the cleaning procedure described by Barker *et al*.^44^. Analyses were performed on a Varian Vista Pro Inductively Coupled Plasma Optical Emission Spectrometer (ICP-OES) and a Perkin Elmer Elan DRCII quadrupole based Inductively Coupled Plasma - Mass Spectrometer (ICP-MS) at the Univ. Cambridge, following established procedures^45^,^46^. Precision for measured Mg/Ca ratios determined from replicate runs of a standard solution containing Mg/Ca = 1.3 mmol/mol was 0.46%. Accuracy of Mg/Ca determinations was confirmed by interlaboratory studies of foraminifera and carbonate reference materials^47^,^48^.

### Analytical methods

Proxy-derivation regressions are y-on-x ordinary least squares regressions on the mean of a fitted gaussian on log-transformed data. An ordinary least squares regression was chosen because the uncertainty in the y-axis variable far exceeds that of the x-axis variable. These histograms are given in the Supplementary material. Volume-predicted PIC:POC has a highly significant relationship with measured PIC:POC (p < 0.001, F-statistic = 123 on 1 and 28 DF), and an excellent linear fit (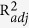 = 0.81). The coccolith aspect ratio (AR = coccolith thickness/*√*coccolith area) is also a highly significant predictor of PIC:POC (p < 0.001, F-statistic = 79 on 1 and 30 DF) and a good linear fit (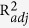 = 0.71).

### Down-core

ODP site 1123 (Expedition 181) is located on Chatham rise, east of New Zealand in the southernmost Pacific (41°47.2′S, 171°29.9′W, 3290 m water depth. The sediment age model for side ODP site 1123 is based on a correlation with the orbitally tuned benthic oxygen isotope stack of LR04^49^,^50^. Reconstructed sea surface temperature (SST) estimates are based on Mg/Ca ratios of planktic forams using species-specific published calibrations. Temperatures across the MIS 7-5 interval were inferred from the *G.bulloides* 250–300 *μ*m and 300–355 *μ*m size fractions using the equation: *Mg*/*Ca* = 0.47 exp 1.08*T*, and those across MIS 15-13 using the equation: *Mg*/*Ca* = 0.299 exp 0.090*T*^51^. [CO_2_(aq)] is estimated from global CO_2_ mixing ratios of an assumed well mixed atmosphere from Vostok and Dome C Antarctic ice cores (Fig. S2; compiled by^52^), using the *seacarb* package in R^53^, with dissolution assumed to be controlled only by SST at a constant salinity of 35. EDC3 gas age was converted to LR04 using a published conversion^54^. Carbon isotopic composition of planktic forams were used as a rough proxy for relative nutrient (~ phosphate) availability corrected for the effect of temperature, using the relationships of 55. See Fig. S3 for time series. Smear slides were prepared using a trial and error approach to attain the optimum coccolith density. SYRACO analysis was carried out in CEREGE.

### SYRACO

In SYRACO analysis, all objects present in the field of view are individually segmented (coccoliths and debris). Secondly the outline of segmented coccoliths is optimized for morphometric measurements. The threshold is computed as the average between the mode of the pixels values (corresponding to the dark background) and the mean of the same segmented image. This threshold value is reproducible and works with very different taxa (*F. profunda*, *E. huxleyi* and *U. sibogae* to a *Sphenolithus* and *Chiasmolithus* etc.). For all images, thresholds and parameters are measured the same way. The area corresponds to the number of the isolated pixels (minus the central are if it exists) multiplied by the scale (area of 1 pixel = 0.0036 *μm*^2^). The mass and the thickness is measured according to a published protocol^20^.

### Effect of PIC:POC on carbonate chemistry

Photosynthesis (net photosynthesis = photosynthesis - respiration) removes one mole of DIC from surface ocean seawater and adds 15/106 moles of alkalinity^56^) for each mole of POC produced. Calcification removes 1 mole of DIC and 2 moles of alkalinity for each mole of PIC produced. [TA] relative to [DIC] dictates the buffering capacity of the surface ocean for CO_2_. [CO_2_] and pH were calculated across DIC and TA values at salinity = 35, temperature = 25 °C, assuming zero concentration of phosphate and silicate and zero hydrostatic pressure, and published values for the first and second dissociation constants of carbonic acid^57^.

## Additional Information

**How to cite this article**: McClelland, H. L. O. *et al*. Calcification response of a key phytoplankton family to millennial-scale environmental change. *Sci. Rep.* **6**, 34263; doi: 10.1038/srep34263 (2016).

## Supplementary Material

Supplementary Information

Supplementary Dataset 1

Supplementary Dataset 2

Supplementary Dataset 3
