# Supplementary material for: Calcification response of a key phytoplankton family to millennial-scale environmental change
Source: Sci Rep. 2016 Sep 28;6:34263. doi: 10.1038/srep34263 (PMC5039703; doi:10.1038/srep34263)
Supplement: Supplementary Information [file srep34263-s1.pdf]

Supplement

Calcification response of a key phytoplankton family to millennial-scale environmental change

H.L.O McClelland[1], N. Barbarin[2], L. Beaufort[2], M. Hermoso[1], P. Ferretti[3,4], M. Greaves[4] & R.E.M. Rickaby[1]

1. Department of Earth Sciences, University of Oxford, South Parks Road, Oxford, OX1 3AN, UK
2. CEREGE CNRS-IRD-Aix Marseille Univ, Avenue Louis Philibert, BP80 13545 Aix en Provence cedex 04, France
3. Consiglio Nazionale delle Ricerche, Istituto per la Dinamica dei Processi Ambientali(CNR-IDPA), Calle, Larga Santa Marta 2137, Venice I-30123, Italy.
4. Godwin Laboratory for Palaeoclimate Research, Department of Earth Sciences, University of Cambridge, Downing Street, Cambridge CB2 3EQ, UK.

Supplementary Discussion

Coccolith to coccosphere relationships

Figure S1 shows the relationships between coccolith dimensions and coccosphere dimensions from our culture experiments. These relationships underpin our rationale. The strong relationship between coccolith length (L_c) and coccosphere diameter (D_s) in the reticulofenestrids is established-Henderiks (2008):

$$D_s = 1.02 + 1.42L_c. \tag{S1}$$

Given that,

$$A_c = \pi \left(\frac{L_c C}{2} \right)^2, \tag{S2}$$

where A_c = coccolith area, W_c and L_c = respectively the semi minor and major axes of the elliptical coccolith and $C = \sqrt{W_c/L_c}$ = "circularity", EqS1 becomes:

$$R_s = 0.51 + \frac{1.42}{\sqrt{\pi}C}\sqrt{A_c}. \tag{S3}$$

When a value of $C = 0.9$ is used, which is typicalHenderiks (2008), the relationship we find between the square root of coccolith area and coccosphere radius is in very close agreement with EqS1. Equation S3 describes the red dashed line in Fig.1C. A more recently published relationship between coccolith length and coccosphere diameter, has a different gradientMüller *et al.* (2010), but the measurements of length are based on coccolith volume, related to distal shield length via an equation from the literatureYoung & Ziveri (2000) - not from direct measurements of area or length. Our direct measurement of and correlation between coccolith dimensions and molar PIC:POC, circumvents the complications associated with allometry and with multiple layers of coccoliths.

Other geological coccolith time series

Some geological time series of coccolith mass from the literature found a decrease in coccolith mass with increasing CO_2Beaufort *et al.* (2011); Meier *et al.* (2014a), whilst others found the oppositeIglesias-Rodriguez *et al.* (2008); Meier *et al.* (2014b). Increasing coccolith mass in the absence of area changes would be expected to correspond to an increase in coccolithophore PIC:POC according to Eq. 3. It is possible that these contrasting conclusions may be reconciled when PIC:POC rather than coccolith mass alone is considered. Unfortunately the data from these studies are not appropriate for this analysis. The earlier version of SYRACO used in these studiesBeaufort *et al.* (2011); Meier *et al.* (2014a,b) yielded significant underestimates of coccolith area, especially for E. huxleyi, and overestimates of coccolith thickness, rendering the estimation of PIC:POC somewhat insensitive to changes in coccolith area. Improvements in the version of SYRACO used in this studyBeaufort *et al.* (2014), are an increased resolution camera, higher magnification lens and images taken in triplicate at different angles to remove the extinction cross. Coccolith volume aloneIglesias-Rodriguez *et al.* (2008) is inappropriate because estimates of PIC:POC using Eq.3 necessitate decoupling volume into thickness and area.

Supplementary figures

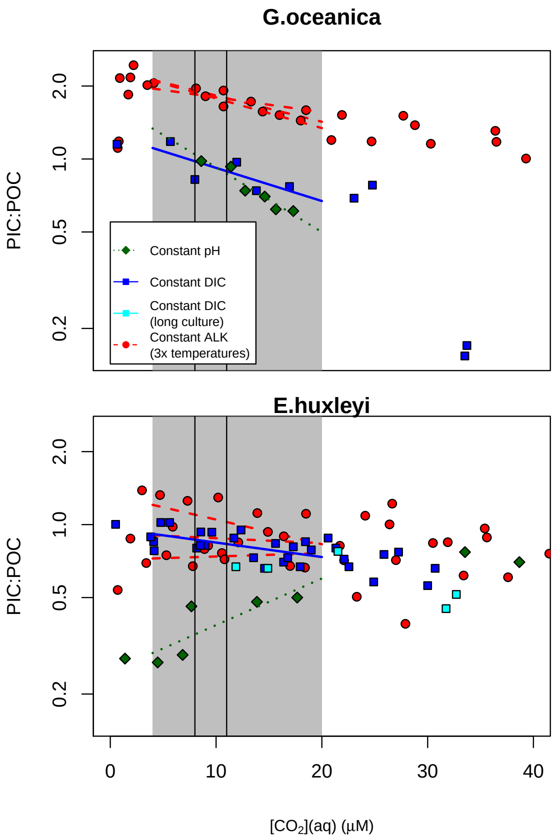

Figure 1: (S1): Plastic response of *E. huxleyi* and *G. oceanica* from the literature. Least-squares linear regressions for each method of carbon manipulation are shown, over a representative range of [CO_2(aq)] (grey shaded region). The solid vertical lines delimit the range of [CO_2(aq)] experienced at ODP site 1123 over the glacial terminations. Three separate regressions are given within the const.ALK experiments, for three experiments undertaken at different temperatures. Slopes are based on the ~4-20μM rage of [CO_2], to capture the linear part of the response, representative of the [CO_2(aq)] range ~8-11μM. *E. huxleyi* data fromBach *et al.* (2011); Iglesias-Rodriguez *et al.* (2008); Langer *et al.* (2009); Müller *et al.* (2010); Sett *et al.* (2014); Zondervan *et al.* (2001) andRiebesell *et al.* (2000), and *G. oceanica* data taken fromRickaby *et al.* (2010); Sett *et al.* (2014); Zondervan *et al.* (2001) and Riebesell *et al.* (2000)). Nb: The constant alkalinity *E. huxleyi* (15°C) data fromBach *et al.* (2011) is the same as that ofSett *et al.* (2014) so has been omitted.

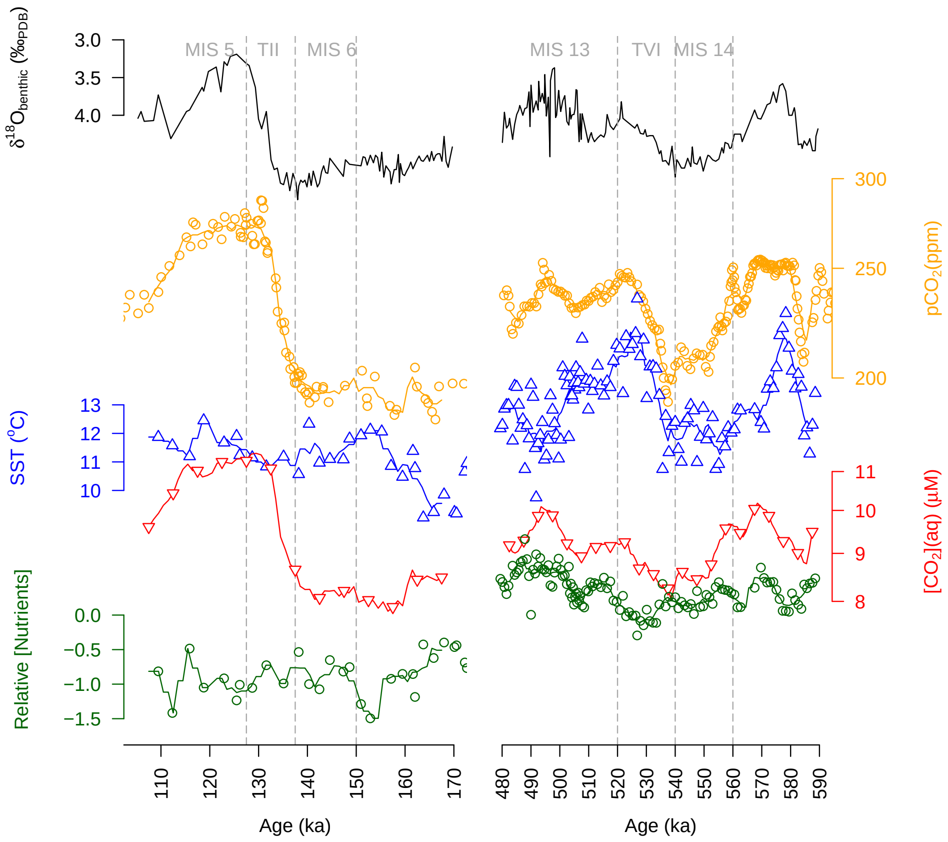

Figure 2: (S2) Time series of proxy-reconstructed climatic parameters at ODP site 1123. Benthic Oxygen isotopes from foramsElderfield *et al.* (2012) allow for temporal alignment with other records. Reconstructed sea surface temperature (SST) estimates are based on Mg/Ca ratios of planktic forams (see methods). [CO_2(aq)] is estimated from global pCO_2 of an assumed well mixed atmosphere from Vostok (*left*) and Dome C (*right*) Antarctic ice cores (compiled byLüthi *et al.* (2008)), with dissolution assumed to be controlled only by SST at a constant salinity of 35. EDC3 gas age was converted to LR04 using a published conversionParrenin *et al.* (2007). Carbon isotopic composition of planktic forams were used as a rough proxy for relative nutrient availability corrected for the effect of temperature (see methods).

Regression data - fitted histograms (Fig.S3 to Fig.S10)

To discount noise from the lower end of some of the mass and area size spectra, which is due to the occasional presence of coccolith fragments, and is an often unavoidable consequence of making smear slides, the values of coccolith morphometrics were found by independently fitting a gaussian curve to each spectrum. The histograms responsible for the data shown in Figure 1B are presented in FigsS4 to S11.

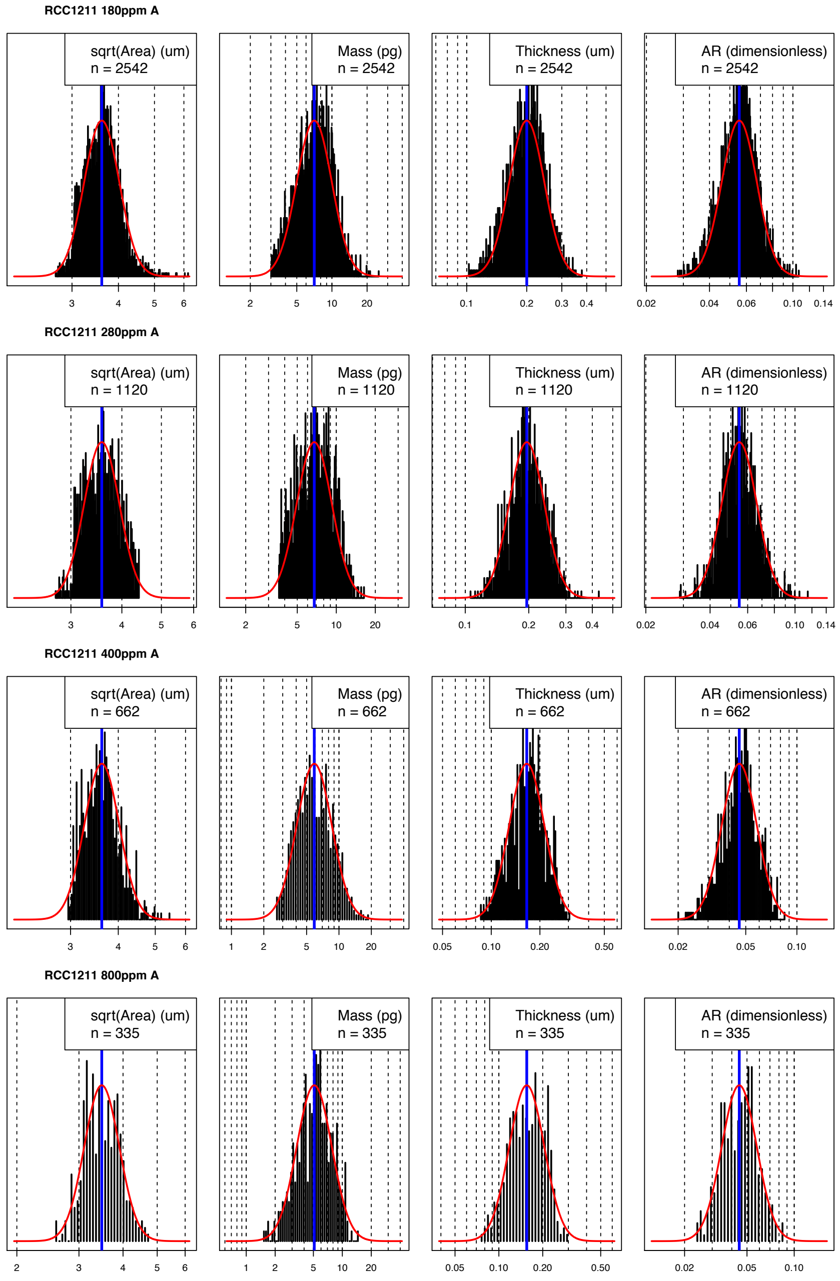

Figure 3: S3: Histograms (1)

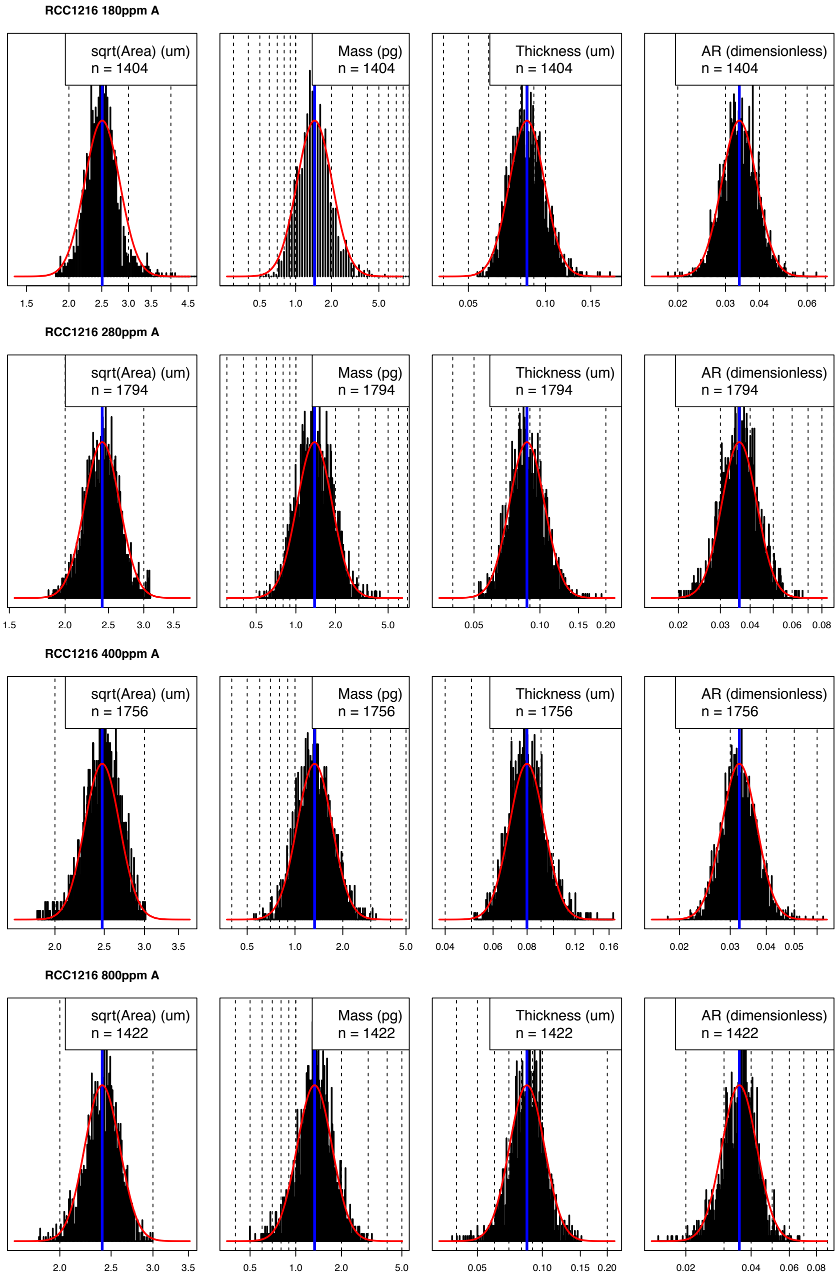

Figure 4: S4: Histograms (2)

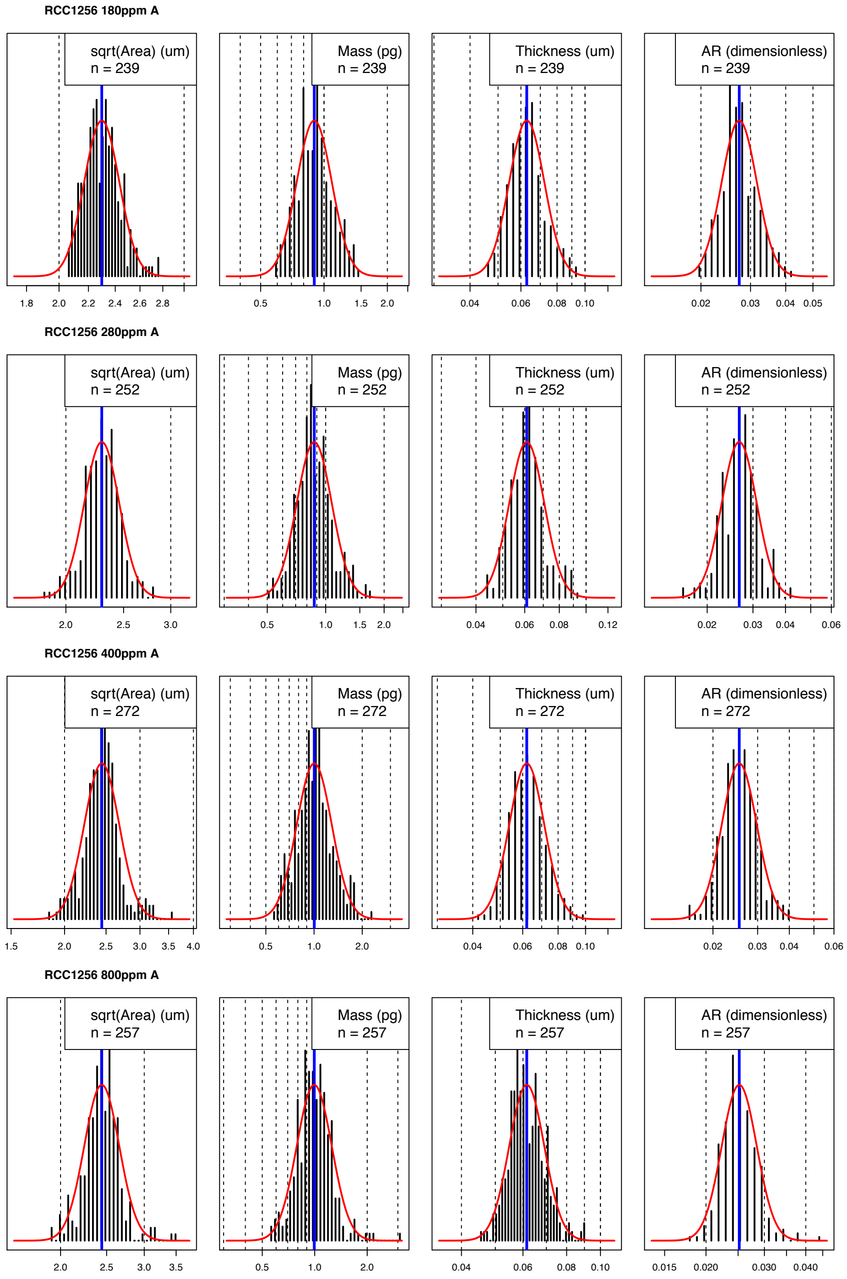

Figure 5: S5: Histograms (3)

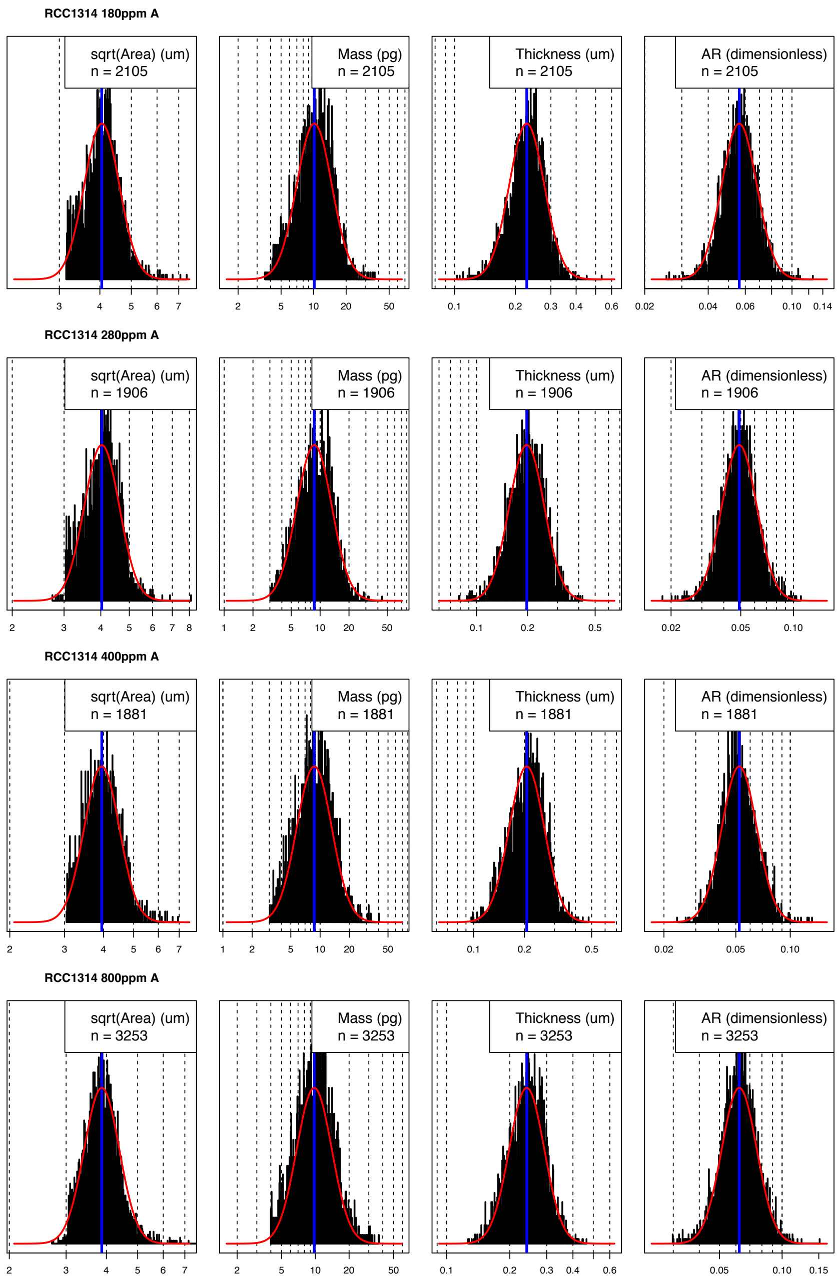

Figure 6: S6: Histograms (4)

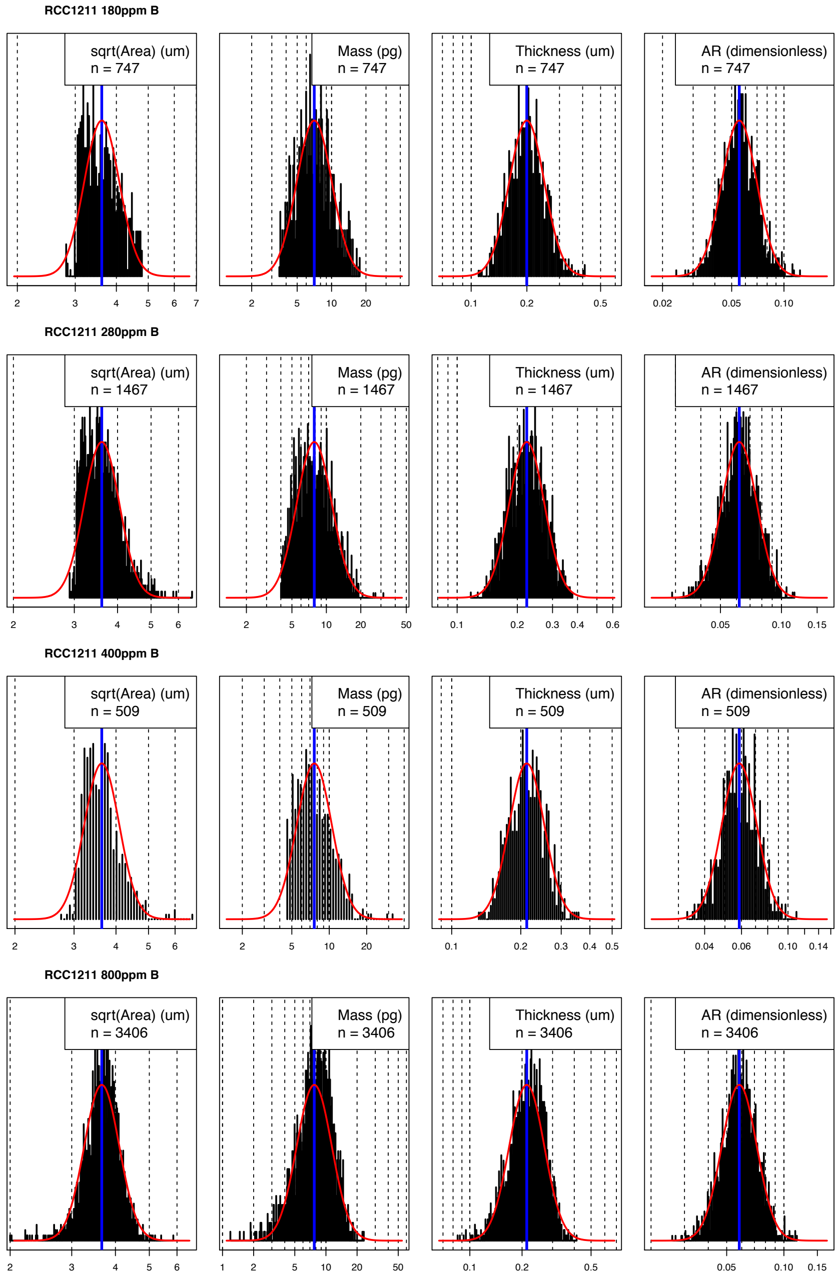

Figure 7: S7: Histograms (5)

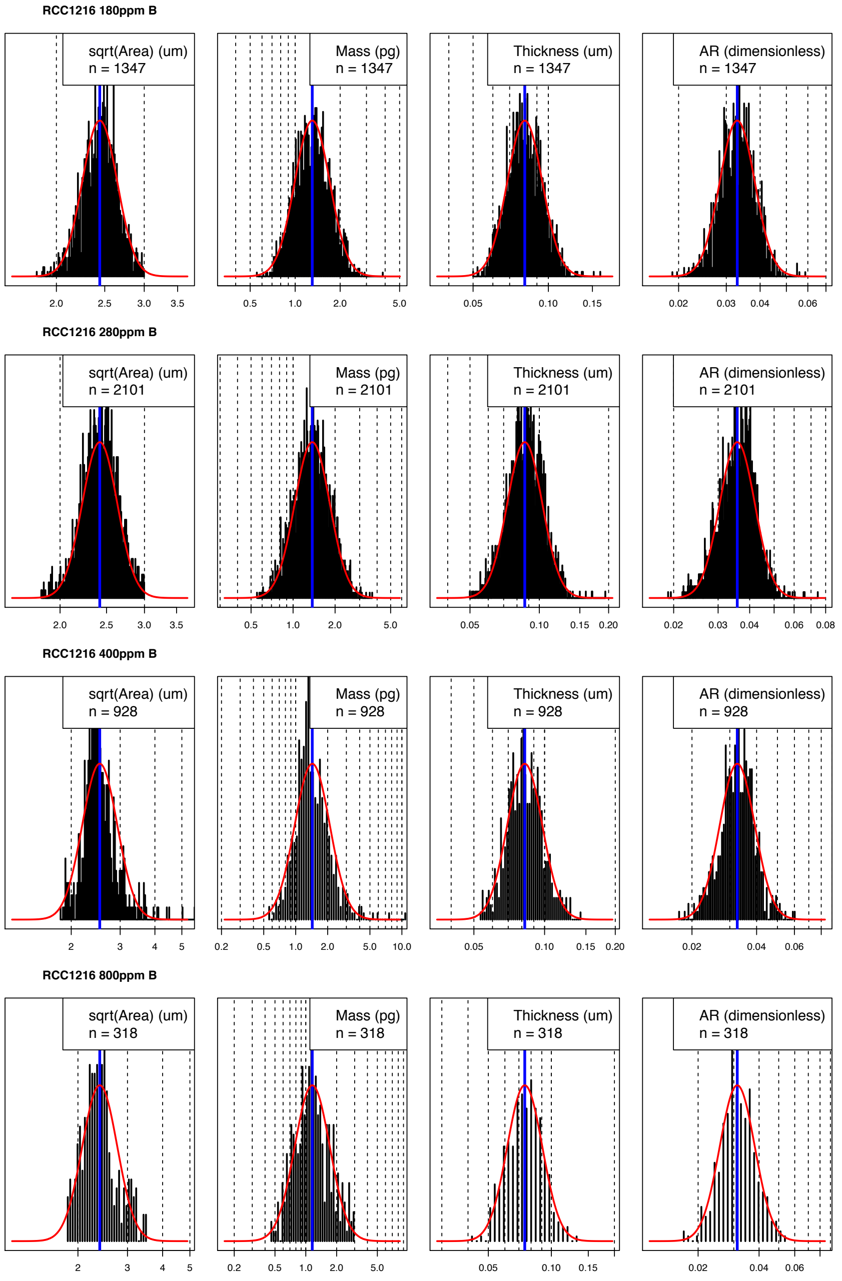

Figure 8: S8: Histograms (6)

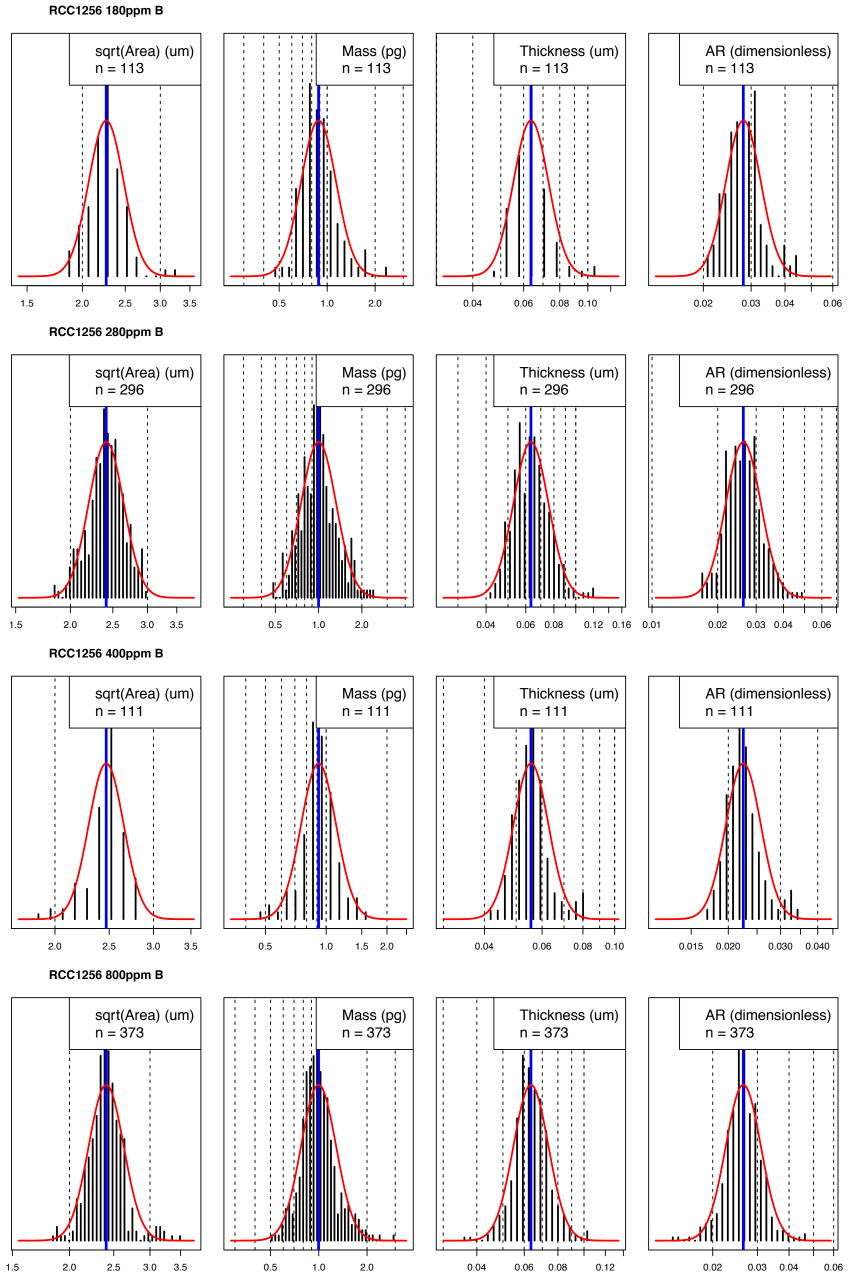

Figure 9: S9: Histograms (7)

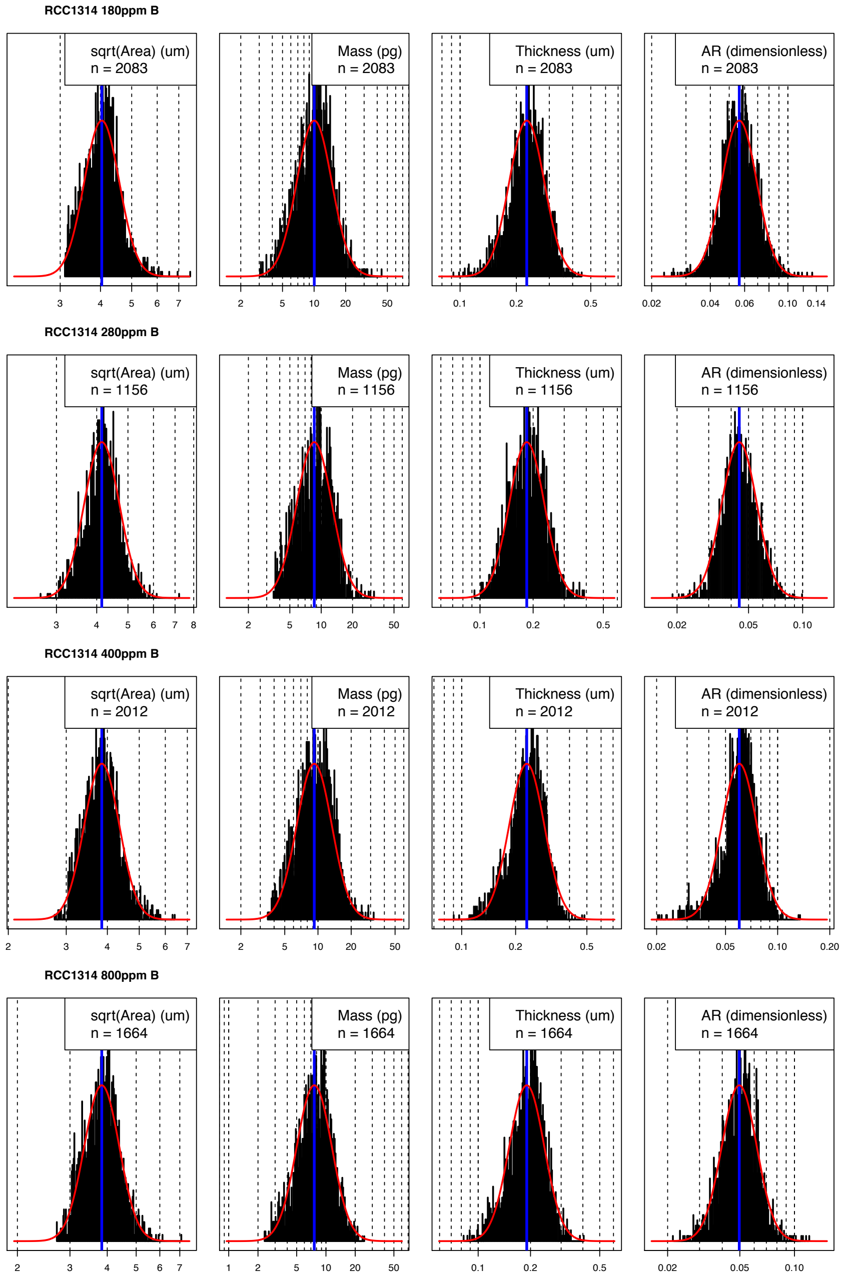

Figure 10: S10: Histograms (8)

Supplementary references

References

Bach, Lennart Thomas, Riebesell, Ulf, & Georg Schulz, Kai. 2011. Distinguishing between the effects of ocean acidification and ocean carbonation in the coccolithophore Emiliania huxleyi. *Limnology and oceanography*, **56**(6), 2040–2050.

Beaufort, L., Probert, I., de Garidel-Thoron, T., Bendif, E. M., Ruiz-Pino, D., Metzl, N., Goyet, C., Buchet, N., Coupel, P., Grelaud, M., Rost, B., Rickaby, R. E. M., & de Vargas, C. 2011. Sensitivity of coccolithophores to carbonate chemistry and ocean acidification. *Nature*, **476**(7358), 80–83.

Beaufort, Luc, Barbarin, Nicolas, & Gally, Yves. 2014. Optical measurements to determine the thickness of calcite crystals and the mass of thin carbonate particles such as coccoliths. *Nature protocols*, **9**(3), 633–42.

Elderfield, H, Ferretti, P, & Greaves, M. 2012. Evolution of Ocean Temperature and Ice Volume Through the Mid-Pleistocene Climate Transition. *Science*, **704**(2012).

Henderiks, Jorijntje. 2008. Coccolithophore size rules Reconstructing ancient cell geometry and cellular calcite quota from fossil coccoliths. *Marine micropaleontology*, **67**(1-2), 143–154.

Iglesias-Rodriguez, M Debora, Halloran, Paul R, Rickaby, Rosalind E M, Hall, Ian R, Colmenero-Hidalgo, Elena, Gittins, John R, Green, Darryl R H, Tyrrell, Toby, Gibbs, Samantha J, von Dassow, Peter, Rehm, Eric, Armbrust, E Virginia, & Boessenkool, Karin P. 2008. Phytoplankton calcification in a high-CO2 world. *Science (new york, n.y.)*, **320**(5874), 336–40.

Langer, G., Nehrke, G., Probert, I., Ly, J., & Ziveri, P. 2009. Strain-specific responses of Emiliania huxleyi to changing seawater carbonate chemistry. *Biogeosciences*, **6**(11), 2637–2646.

Lüthi, Dieter, Le Floch, Martine, Bereiter, Bernhard, Blunier, Thomas, Barnola, Jean-Marc, Siegenthaler, Urs, Raynaud, Dominique, Jouzel, Jean, Fischer, Hubertus, Kawamura, Kenji, & Stocker, Thomas F. 2008. High-resolution carbon dioxide concentration record 650,000-800,000 years before present. *Nature*, **453**(7193), 379–82.

Meier, K. J. S., Beaufort, L., Heussner, S., & Ziveri, P. 2014a. The role of ocean acidification in Emiliania huxleyi coccolith thinning in the Mediterranean Sea. *Biogeosciences*, **11**(10), 2857–2869.

Meier, K.J.S., Berger, C., & Kinkel, H. 2014b. Increasing coccolith calcification during CO2 rise of the penultimate deglaciation (Termination II). *Marine micropaleontology*, **112**(July), 1–12.

Müller, M. N., Schulz, K. G., & Riebesell, U. 2010. Effects of long-term high CO2 exposure on two species of coccolithophores. *Biogeosciences*, **7**(3), 1109–1116.

Parrenin, F, Barnola, J, Beer, J, Blunier, T, Castellano, E, Chappellaz, J, Dreyfus, G, Fischer, H, & Fujita, S. 2007. of the Past The EDC3 chronology for the EPICA Dome C ice core. 485–497.

Rickaby, R. E. M., Henderiks, J., & Young, J. N. 2010. Perturbing phytoplankton: response and isotopic fractionation with changing carbonate chemistry in two coccolithophore species. *Climate of the past*, **6**(6), 771–785.

Riebesell, Ulf, Zondervan, Ingrid, Rost, B.È., Tortell, P.D., Zeebe, R.E., & Morel, F.Ë.M.M. 2000. Reduced calcification of marine plankton in response to increased atmospheric CO2. *Nature*, **407**(September), 2–5.

Sett, Scarlett, Bach, Lennart T, Schulz, Kai G, Koch-Klavsen, Signe, Lebrato, Mario, & Riebesell, Ulf. 2014. Temperature Modulates Coccolithophorid Sensitivity of Growth, Photosynthesis and Calcification to Increasing Seawater pCO2. *Plos one*, **9**(2), e88308.

Young, Jeremy R., & Ziveri, Patrizia. 2000. Calculation of coccolith volume and it use in calibration

of carbonate flux estimates. *Deep sea research part ii: Topical studies in oceanography*, **47**(9-11), 1679–1700.

Zondervan, I., Zeebe, R.E., Rost, B., & Riebesell, U. 2001. Decreasing marine biogenic calcification: A negative feedback. *Global biogeochemical cycles*, **15**(2), 507–516.
